# A highly durable fuel cell electrocatalyst based on double-polymer-coated carbon nanotubes

**DOI:** 10.1038/srep16711

**Published:** 2015-11-23

**Authors:** Mohamed R. Berber, Inas H. Hafez, Tsuyohiko Fujigaya, Naotoshi Nakashima

**Affiliations:** 1 International Institute for Carbon Neutral Energy Research (WPI-I2CNER), Kyushu University, 744 Motooka, Nishi-ku, Fukuoka 819-0395 (Japan); 2 Department of Chemistry, Faculty of Science, Tanta University, Tanta 31527 (Egypt); 3 Department of Natural resources and Agricultural Engineering, Faculty of Agriculture, Damanhour University, Damanhour 22516 (Egypt); 4 Department of Applied Chemistry, Graduate School of Engineering, Kyushu University, 744 Motooka, Nishi-ku, Fukuoka 819-0395 (Japan); 5 JST-CREST, 5 Sanbancho, Chiyoda-ku, Tokyo, 102-0075 (Japan)

## Abstract

Driven by the demand for the commercialization of fuel cell (FC) technology, we describe the design and fabrication of a highly durable FC electrocatalyst based on double-polymer-coated carbon nanotubes for use in polymer electrolyte membrane fuel cells. The fabricated electrocatalyst is composed of Pt-deposited polybenzimidazole-coated carbon nanotubes, which are further coated with Nafion. By using this electrocatalyst, a high FC performance with a power density of 375 mW/cm^2^ (at 70 ˚C, 50% relative humidity using air (cathode)/H_2_(anode)) was obtained, and a remarkable durability of 500,000 accelerated potential cycles was recorded with only a 5% loss of the initial FC potential and 20% loss of the maximum power density, which were far superior properties compared to those of the membrane electrode assembly prepared using carbon black in place of the carbon nanotubes. The present study indicates that the prepared highly durable fuel cell electrocatalyst is a promising material for the next generation of PEMFCs.

we describe the design and fabrication of a highly durable fuel cell electrocatalyst based on double layer-polymer-coated carbon nanotubes as the catalyst support. The polymers used for the double layer-coating are polybenzimidazole (PBI) and Nafion. The fabricated polymer electrolyte membrane fuel cell (PEMFC) catalyst showed only a 5% loss of the initial fuel cell potential and 20% loss of the maximum power density even after a 500,000-cycle durability test between 1.0 and 1.5 V.

Since oil, as the main source of energy, will soon be limited, there is a social demand to find an alternative sustainable green source of energy with a high power density. One of the most promising alternative energy technologies is fuel cells (FCs), in particular, PEMFCs that provide an instant power output with a high energy conversion efficiency and are CO_2_ emission-free^1^. PEMFCs have received a great deal of attention for practical use in housing, transportation and portable electronic devices^2^. Electrode catalysts composed of platinum (Pt)/carbon black (CB)/ionomer (typically Nafion) have been widely used in many studies^3^,^4^, and much effort has been devoted to the design and fabrication of a high performance and durable electrocatalysts for PEMFCs^5^. It has been reported that the ideal PEMFC electrocatalyst should have a high electrical conductivity, excellent catalytic activity especially for oxygen reduction reaction, a high surface area, and a high corrosion resistance as well as low cost. In addition, it should carry a high proton conductivity to enhance smooth electrode reactions^6^.

Multi-walled carbon nanotubes (MWNTs) have received broad attention as a FC catalyst support because they have a high electrical conductivity, a large surface area, a highly crystalline structure, and high electrochemical stability, which are important to the FCs with for high durability^7^,^8^,^9^. However, MWNTs with highly crystalline structures lack binding sites for homogeneous loading of the metal catalysts, e.g., Pt metal nanoparticles (Pt-NPs). Thus, they need to be functionalized before their use in FC catalysts. Chemical oxidation of the MWNTs with strong acids is a conventional activation technique for MWNTs to create surface functional groups, such as oxygen containing groups, e.g., C=O and COOH, for homogeneous Pt-NP loading^10^. However, such an oxidation technique sacrifices the pristine MWNT structure, resulting in fast degradation of the prepared electrocatalyst during FC operating conditions^11^. Thus, an alternative functionalization strategy to utilize the pristine MWNT structure is necessary because the pristine structure of the MWNTs is an important factor for the FC durability. Recently, we developed a polymer-coating technique for MWNTs; specifically, pristine MWNTs were wrapped with poly[2,2′-(2,6-pyridine)-5,5′-bibenzimidazole] (PyPBI) (Fig. 1a), in which the polymer worked as a MWNT-solubilizer as well as a binder for homogeneous loading of the Pt-NPs^12^,^13^. The prepared catalyst showed an efficient FC performance under non-humidified conditions at 120 °C^14^.

It is well known that not only the proton conductivity of the membranes, but also that of the electrocatalyst strongly affects FC performance since it is essential to form a triple phase structure. A principle strategy to improve the proton conductivity of the FC electrocatalyst is to cast an ionomer onto the surface of the electrocatalyst^15^. At present, perfluorosulfonate as an acidic ionomer with a high proton conductivity, such as that used in Nafion^16^,^17^, has been widely used to improve the proton conductivity of the membrane electrode assembly (MEA)^18^,^19^,^20^. However, pristine MWNTs are unable to provide good binding sites for sufficient attachment of such an ionomer. Thus, when using such an ionomer, it leaches from the MEA during long term FC operating conditions, resulting in a lower FC performance^21^. Very recently, we developed a novel technique of improving the proton conductivity of the FC electrocatalyst^22^, in which (PyPBI)-wrapped MWNTs were further coated with a proton conducting ionomer, polyvinylphosphonic acid (PVPA), through an acid-base reaction between PyPBI and PVPA. The high temperature (120 °C) FC performance and durability results of that system were promising due to the improvement of the catalyst proton conductivity and stability.

In the present study, we introduce our developed “double polymer-coating” technique to conventional FC systems that are operated at low temperatures (~70 °C) under humidified conditions using Nafion (Fig. 1b,c). Specifically, a Nafion ionomer was used to further coat the PyPBI-coated MWNTs as the 2^nd^ layer-polymer via an acid-base interaction^23^. The Nafion ionomer was added to support a good proton transfer and to improve the triple phase boundary structure of the electrocatalyst, and accordingly facilitates a good oxygen reduction reaction (see the schematic illustration of Fig. 1d). In order to clarify the importance of the MWNTs as a catalyst support, another electrocatalyst using CB was synthesized (Fig. 1c). We then fabricated MEAs (for a photo image, see Supplementary Fig. 1) using the prepared electrocatalysts and a Nafion® 117 membrane. The FC performance of the assembled MEAs was measured at 70 °C under a relative humidity of ~50%. The durability of the fabricated MEAs was evaluated using a potential-dynamic protocol, which is more closely related to the drive-cycle operation of the PEMFCs in vehicles (see Supplementary Fig. 2). Subsequently, the results of the double polymer coating system of both the MWNT-based and CB-based electrocatalysts were compared with those of the commercial CB-based catalyst that is composed of the CB, Pt and Nafion (CB/Pt/Nafion), and measured under the same FC and durability conditions. In this study, we also describe the possible reasons of the prolonged durability for the MWNT/PyPBI/Pt/Nafion-based MEA.

## Results

The Pt-NPs loaded on PyPBI-wrapped MWNTs (MWNT/PyPBI/Pt) and CB (CB/PyPBI/Pt) were prepared according to our previous reports^22^,^24^. The X-ray photoelectron spectroscopy (XPS) spectra were almost identical to that of the reported ones (see Supplementary Figs 3 and 4). Figure 2a shows the XPS spectra (survey scan) of the MWNT/PyPBI/Pt (purple), CB/PyPBI/Pt (black) and CB/Pt (gray)^25^ after the Nafion treatment. As can be seen, the spectra of both the Nafion-treated MWNT/PyPBI/Pt (MWNT/PyPBI/Pt/Nafion) (purple) and Nafion-treated CB/PyPBI/Pt (CB/PyPBI/Pt/Nafion) (black) show the characteristic peaks of Nafion such as F_1s_ at 689 eV, while no Nafion peak was recognized on the Nafion-treated CB/Pt (gray); namely, the narrow scans of the F_1s_ region (Supplementary Fig. 5) indicated the absence of Nafion on the Nafion-treated commercial-CB/Pt composite obtained after rinsing with methanol and 2-propanol. These results demonstrate the importance of PyPBI (a basic polymer) as a binder to attach the Nafion (an acidic polymer) on the PBI-coated MWNTs and PBI-coated CB^23^. When comparing the S_2p_ spectrum of the Nafion-coated MWNT/PyPBI/Pt to that of the free Nafion (Fig. 2b), a shift in the binding to a lower energy value by 2.5 eV is observed; this would be due to the interaction between the sulfonic group of Nafion and nitrogen groups of PyPBI, where a similar observation was reported for the polyaniline-modified poly(styrene sulfonate) membranes^26^. Also of importance is the N_1s_ peak (Fig. 2c); namely, we recognized a new peak appeared around 401.6 eV that is attributed to the NH^+^ group on the MWNT/PyPBI/Pt/Nafion, supporting the interaction of the PyPBI with the SO_3_^–^ moieties of Nafion^27^,^28^,^29^. We have also carried out FT-IR measurements of the MWNT/PyPBI/Pt before and after the Nafion coating to confirm the chemical interaction between the PyPBI and Nafion (see results and discussion in Supplementary Fig. 6).

Figure 2d shows the thermogravimetric analysis (TGA) curves of the MWNT/PyPBI/Pt (orange) and MWNT/PyPBI/Pt/Nafion (purple) together with the differential thermogravimetric analysis (DTGA) of the MWNT/PyPBI/Pt/Nafion (black). The weight-reduction of ca. 3 wt% at temperatures up to 200 °C that appeared in the MWNT/PyPBI/Pt/Nafion is attributed to the physically-adsorbed water. The first stage of the Nafion degradation and decomposition is known to be initiated at 280 °C^30^. The weight loss observed in the 280–390 °C interval is attributed to the loss of the SO_2_ and CO_2_ species of the Nafion (purple line in Fig. 2d)^30^. The weight-loss observed in the range of 400–450 °C is attributed to the loss of the PyPBI moieties (orange line in Fig. 2d)^31^. The loss observed above 450 °C is attributed to the decomposition of the MWNTs and the C-F fragments of Nafion^32^. The apparent amount of Pt determined from the weight residue at 900 °C decreased from 44.1 to 38.0 wt% after the Nafion coating. Therefore, the Nafion content in the electrocatalyst was determined to be 16.0 wt% (for more details about the calculations, see Supplementary information). On the other hand, the TGA curves of the CB/PyPBI/Pt (gray) and CB/PyPBI/Pt/Nafion (black) showed similar results (see Supplementary Fig. 7); namely, the apparent amount of the Pt determined from the weight residue at 900 °C decreased from 43.3 to 37.8 wt% after the Nafion coating. Therefore, the Nafion content in the CB/PyPBI/Pt/Nafion was determined to be 14.6 wt%. The amount of Pt loading on the CB/Pt (Nafion-treated) determined from the TGA residue was 41.0% (see Supplementary Fig. 8). Figure 3a shows the transmission electron microscopy (TEM) image of the MWNT/PyPBI/Pt/Nafion, in which Pt-NPs of 4.1 nm with a narrow Pt particle size distribution (Fig. 3b) were homogeneously deposited on the nanotubes. This result agreed with the previously reported TEM image of MWNT/PyPBI/Pt^22^. The TEM image of CB/PyPBI/Pt/Nafion (Fig. 3c) showed similar results with a narrow Pt-particle size distribution (Fig. 3d, 3.7 ± 0.4 nm). For comparison, the TEM image and the particle size distribution of the commercial CB/Pt (2.9 ± 0.4 nm) are shown in Fig. 3e,f, respectively.

The electrochemical surface area (ECSA) results of the prepared electrocatalysts, as determined from the cyclic voltammogram (CV) measurements (Fig. 4a), were 59.5 and 62.0 m^2^/g_Pt_ for the MWNT/PyPBI/Pt/Nafion (purple) and CB/PyPBI/Pt/Nafion (black), respectively. The slight increase in the ECSA of CB/PyPBI/Pt/Nafion would be due to the smaller Pt-NPs on the surfaces of the CB (see Fig. 3c)^33^. For comparison, the gray spectrum of Fig. 4a shows the CV of the commercial CB/Pt (Nafion-treated). The ECSA of this catalyst was calculated to be 64.5 m^2^/g_Pt_.

Figure 4b displays the polarization (dashed) and power density (solid) curves of the MWNT/PyPBI/Pt/Nafion-based MEA (purple) and CB/PyPBI/Pt/Nafion-based MEA (black). As can be seen in Fig. 4b, the open circuit voltages (OCV) of the two different MEAs were almost 1.0 V, reflecting the good assembly of the anode and cathode electrodes and the Nafion membrane. The MWNT/PyPBI/Pt/Nafion-based MEA exhibited much higher power density and current density compared to that of the CB/PyPBI/Pt/Nafion-based MEA. For comparison, the gray curves of Fig. 4b show the polarization (dashed) and the power density (solid) curves of the CB/Pt-based MEA (for CB/Pt-based MEA, the same amount of Nafion found in CB/PyPBI/Pt/Nafion composite was added). As seen, the FC performance of the CB/Pt-based MEA was almost similar to that of the CB/PyPBI/Pt/Nafion-based MEA.

In order to explore the reason of the difference of FC performance, electrochemical impedance spectral (EIS) measurements were carried out. Figure 4c shows the Nyquist plots of the MWNT/PyPBI/Pt/Nafion-based MEA (purple) and CB/PyPBI/Pt/Nafion-based MEA (black). The equivalent circuit used to fit the obtained impedance spectra is shown in Fig. 4d. In Fig. 4c, each spectrum exhibits two overlapped loops corresponding to charge transfer resistance (R_ct_) and gas diffusion resistance (R_g_) in the high and low frequency regions, respectively^34^. The high frequency intercept at the real impedance axis represents the ohmic resistance of the cell (R_Ω_)^35^. Table 1 summarizes the obtained R_Ω_, R_ct_ and R_g_ resistances, in which we see a greater decrease in the total impedance of the MWNT/PyPBI/Pt/Nafion-based MEA (564 mOhm cm^2^) compared to that of the CB/PyPBI/Pt/Nafion-based MEA (976 mOhm cm^2^). Especially, the R_ct_ of the MWNT/PyPBI/Pt/Nafion-based MEA (32 mOhm cm^2^) showed a low value compared to that of the CB/PyPBI/Pt/Nafion-based MEA (287 mOhm cm^2^), indicating a smoother charge transfer during the fuel cell operation of the MWNT/PyPBI/Pt/Nafion-based MEA. In addition, we see a remarkable decrease in the R_g_ value for the MWNT/PyPBI/Pt/Nafion-based MEA (532 mOhm cm^2^) compared to that of the CB/PyPBI/Pt/Nafion-based MEA (689 mOhm cm^2^) probably due to the superior morphology of MWNTs having a fibrous network structure, which is advantageous for gas diffusion, while CB has an agglomerative structure that might work as a slight barrier to fuel gas access^36^.

The SEM images of both MWNT/PyPBI/Pt/Nafion-based electrode and CB/PyPBI/Pt/Nafion-based electrode (Supplementary Fig. 9) clearly showed the morphology difference between the two carbon supports (MWNTs and CB), confirming the advantageous use of MWNTs as a carbon support for enhancing the charge transfer and gas diffusion. Based on these results, a schematic model for the structure of the gas diffusion electrode of the MWNT/PyPBI/Pt/Nafion-based MEA is illustrated in Supplementary Fig. 10, in which the Nafion ionomer is homogeneously distributed around the PyPBI-wrapped MWNTs, providing a good proton-conducting path as well as the formation of an ideal triple phase boundary structure that is suitable for smooth electrochemical reactions.

To investigate the stability of the newly fabricated electrocatalyst, accelerated stress durability testing was performed. Figure 5 shows the polarization (A and C) and power density (B and D) curves of the MWNT/PyPBI/Pt/Nafion-based MEA (A and B) and the CB/PyPBI/Pt/Nafion-based MEA (C and D), respectively. For comparison, the polarization (A) and power density (B) curves of the commercial CB/Pt-based MEA are measured using the same durability protocol and are displayed in the Supplementary Fig. 11. The cell voltage at 200 mAcm^–2^ (Fig. 6a) and the maximum power density (Fig. 6b) of the MWNT/PyPBI/Pt/Nafion-based MEA (purple plots), CB/PyPBI/Pt/Nafion-based MEA (red plots) and CB/Pt-based MEA (green plots), plotted as a function of the potential cycling numbers, are shown in Fig. 6. The value of 200 mAcm^–2^ was chosen since this current density has been used for stationary polymer electrolyte FCs^37^. The cell voltage of the MWNT/PyPBI/Pt/Nafion-based MEA showed a very low degradation rate compared to that of the CB/PyPBI/Pt/Nafion-based MEA; namely, the FC performance of the MWNT/PyPBI/Pt/Nafion-based MEA was active even after the 500,000 cycle-durability test (~5% loss of the initial voltage at 200 mAcm^–2^ was observed; Fig. 6a, purple plot). Also, the maximum power density (initial value 375 mWcm^–2^) of the MEA showed only a 20% decrease after this high number of potential-dynamic cycles (Fig. 6b, purple plot).

In contrast, the CB/PyPBI/Pt/Nafion-MEA showed a 15% loss in the initial cell voltage at 200 mAcm^–2^ and 43% loss of the maximum power density (initial value of 200 mWcm^–2^) after 100,000 potential-dynamic cycles (Fig. 6a,b, respectively, red plots). Although CB/PyPBI/Pt/Nafion-MEA showed lower durability than MWNT/PyPBI/Pt/Nafion-MEA, both MEAs showed a much higher durability than commercial CB/Pt-based MEA, which shows a 50% loss of the initial cell potential at 200 mAcm^–2^ and 65% loss of the maximum power density after only 10,000 potential-dynamic cycles (Fig. 6a,b, respectively, green plots). Supplementary Table 1, summarizes the data of the FC performance of the fabricated electrocatalysts.

To investigate the reasons of the difference of the durability, the MEAs after the durability test were delaminated and analyzed. The TEM image of the MWNT/PyPBI/Pt/Nafion-based electrocatalyst collected from the cathode sides is shown in Fig. 7a. We observed that Pt-NPs with a diameter of 6.6 ± 0.9 nm (see Fig. 7b) which is slightly larger than the initial diameter (4.1 ± 0.5 nm) are still well-distributed on the carbon nanotube support, whereas the morphology of MWNTs was not very different from the original one. The CB/PyPBI/Pt/Nafion-based electrocatalyst has showed a much higher increase of Pt-NPs from 3.7 ± 0.4 nm before durability to 10.9 ± 1.8 nm after durability (see Fig. 7c,d). In sharp contrast, the commercial CB/Pt-based electrocatalyst showed very few Pt-NPs with a large bare carbon support area after the durability test (Fig. 7e,f), indicating a the loss of the Pt-NPs.

In order to obtain information about the degree of the oxidation of the MWNTs after the durability test, Raman measurements were carried out by monitoring the intensity of D-band (I_D_) and G-band (I_g_), and the results of the MWNT/PyPBI/Pt/Nafion before (solid lines) and after (dashed lines) the durability test are shown in Fig. 8. As seen from the figure, only small change in the (I_D_/I_G_)^38^ ratio (from 0.62 to 0.95 after the durability test) was observed, which reflects the structural stability of the MWNTs^39^. Accordingly, this result emphasizes the stability of the MWNT which contributed to the observed remarkably high durability of the MWNT/PyPBI/Pt/Nafion-based MEA.

## Discussion

**Figure 1:**
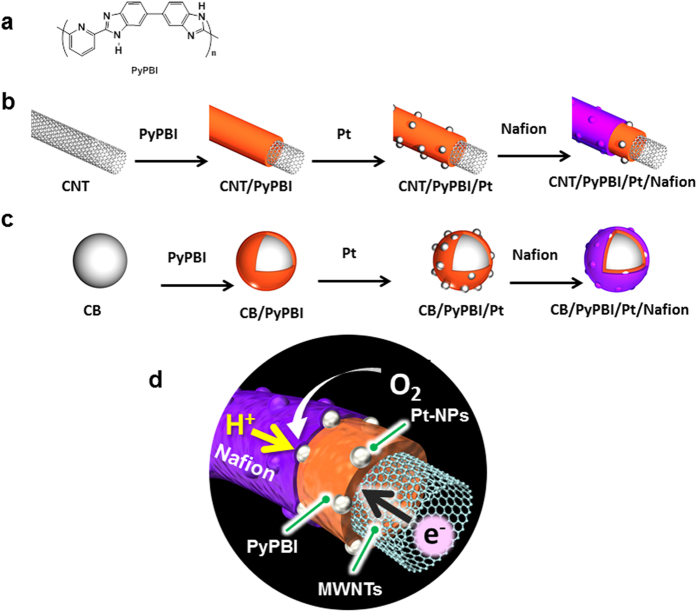
Schematic illustrations. (**a**) Chemical structure of PyPBI. (**b**,**c**) Schematic illustrations for the preparation of MWNT/PyPBI/Pt/Nafion (**b**) and CB/PyPBI/Pt/Nafion (**c**).

**Figure 2:**
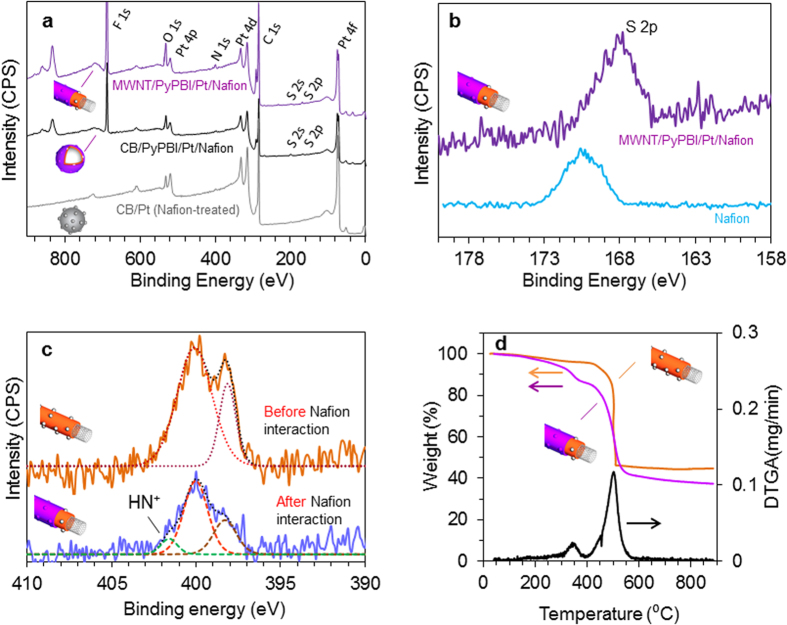
XPS and TGA analyses. (**a**) XPS survey spectra after Nafion coating of the MWNT/PyPBI/Pt (purple spectrum), CB/PyPBI/Pt (black spectrum) and CB/Pt (gray spectrum). (**b**) XPS core spectrum of S_2p_ of Nafion (blue), and Nafion-coated MWNT/PyPBI/Pt (purple). (**c**) XPS core spectrum of N_1s_ of the MWNT/PyPBI/Pt before (orange) and after (purple) Nafion coating. (**d**) TGA curves of the MWNT/PyPBI/Pt (orange) and MWNT/PyPBI/Pt/Nafion (purple). Differential thermal analysis (DTGA) of the MWNT/PyPBI/Pt/Nafion is represented by the black spectrum.

**Figure 3:**
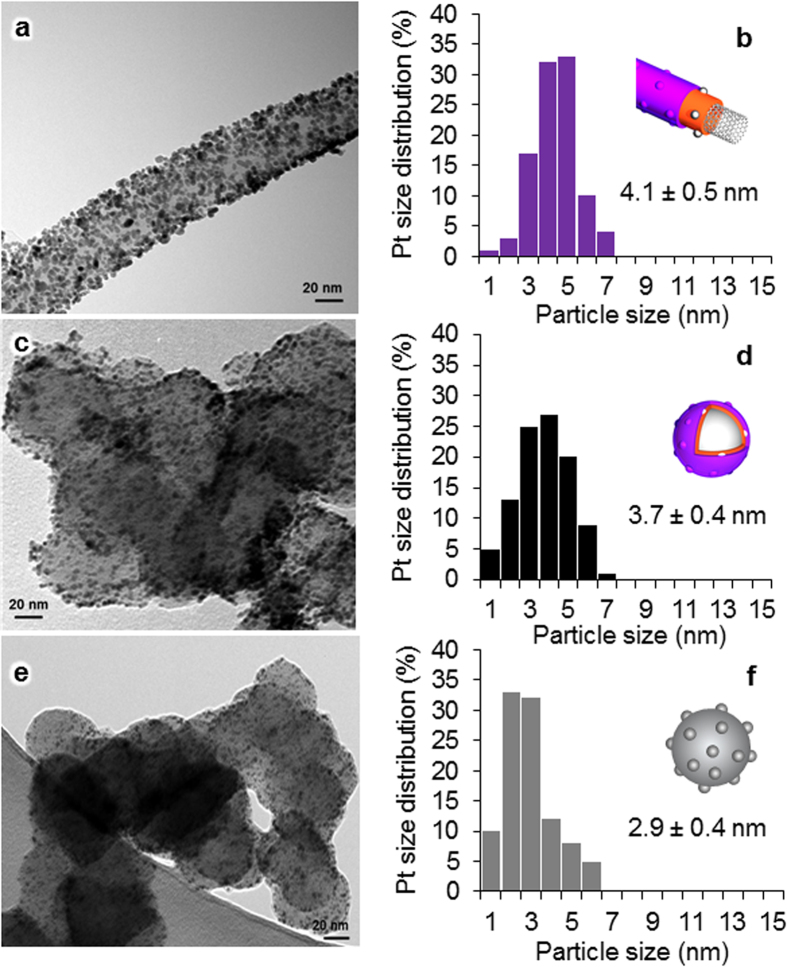
TEM images and Pt-size distribution. TEM image and Pt-size distribution of MWNT/PyPBI/Pt/Nafion (**a**,**b**, respectively), CB/PyPBI/Pt/Nafion (**c**,**d**, respectively), and CB/Pt composite treated with Nafion (**e**,**f**, respectively), before durability test.

A new-type of FC electrocatalyst was synthesized using pristine MWNTs and CB supports, which are first wrapped with PyPBI (basic polymer) on which Pt-NPs were deposited and then further coated with Nafion (acidic polymer) *via* the acid-base reaction between Nafion and PyPBI to obtain the MWNT/PyPBI/Pt/Nafion and CB/PyPBI/Pt/Nafion as shown in Fig. 1b,c. The prepared catalysts were characterized using XPS (Fig. 2a–c), TGA (Fig. 2d), TEM (Fig. 3), Raman (Fig. 8), and FTIR (Supplementary Fig. 6), confirming the designed structures as shown in Supplementary Fig. 1.

**Figure 4:**
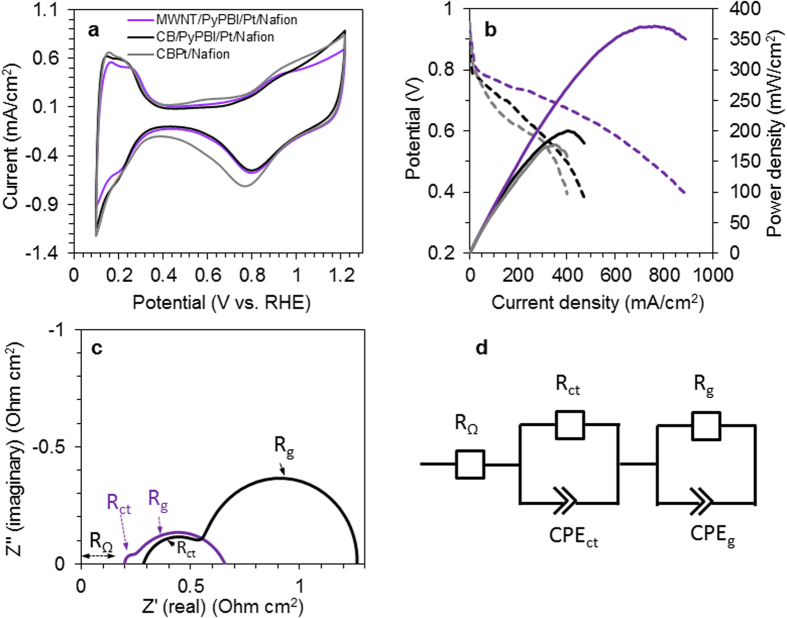
Cyclic voltammetry, fuel cell, and impedance analyses. (**a**) CV of MWNT/PyPBI/Pt/Nafion (purple), CB/PyPBI/Pt/Nafion (black), and CB/Pt/Nafion-treated (gray). (**b**) Polarization (dashed) and power density (solid) curves of the MWNT/PyPBI/Pt/Nafion-based MEA (purple), CB/PyPBI/Pt/Nafion-based MEA (black), and CB/Pt/Nafion-treated (gray). (**c**) Nyquist plots of MWNT/PyPBI/Pt/Nafion-based MEA (purple) and CB/PyPBI/Pt/Nafion-based MEA (black). (**d**) Equivalent circuit used to fit the obtained impedance spectra.

**Figure 5:**
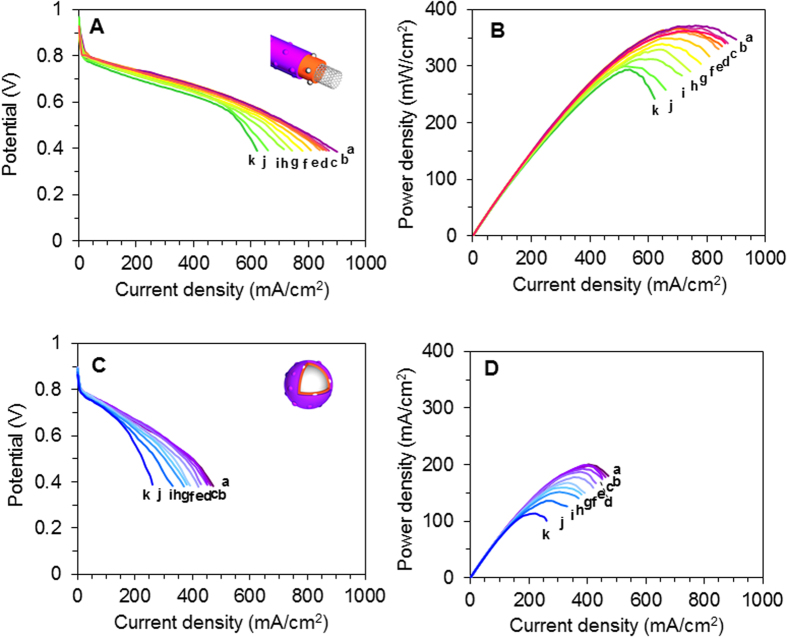
Durability test. Polarization curves (**A**,**C**) and power density curves (**B**,**D**) during durability testing of: the MWNT/PyPBI/Pt/Nafion-based MEA (**A**,**B**) and the CB/PyPBI/Pt/Nafion-based MEA (**C**,**D**). For simplicity, some representative curves are selected for display. For MWNT/PyPBI/Pt/Nafion-based MEA (**A**,**B**), the Arabic letters, a, b, c, d, e, f, g, h, I, j and k, represent the performance after 1,000, 50,000, 100,000, 150,000, 200,000, 250,000, 300,000, 350,000, 400,000, 450,000, and 500,000 potential cycles, respectively. For CB/PyPBI/Pt/Nafion-based MEA (**C**,**D**), the Arabic letters a, b, c, d, e, f, g, h, I, and j represent the performance after 10,000, 20,000, 30,000, 40,000, 50,000, 60,000, 70,000, 80,000, 90,000, and100,000 potential cycles, respectively.

**Figure 6:**
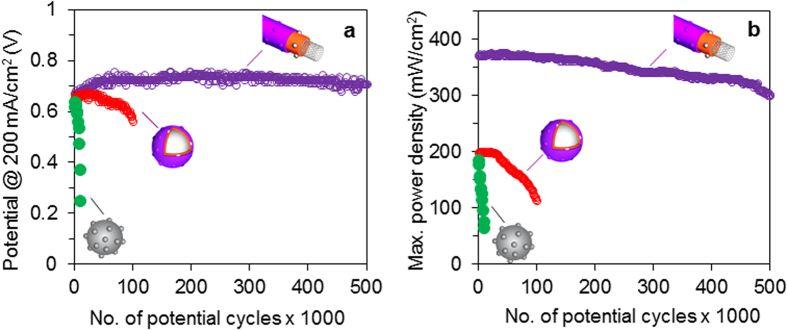
FC performance during durability tests. Cell voltage at 200mA.cm^−2^ (**a**) and maximum power density (**b**) as a function of potential cycling numbers of: MWNT/PyPBI/Pt/Nafion-based MEA (purple), CB/PyPBI/Pt/Nafion-based MEA (red), and CB/Pt-based MEA treated with Nafion (green).

**Figure 7:**
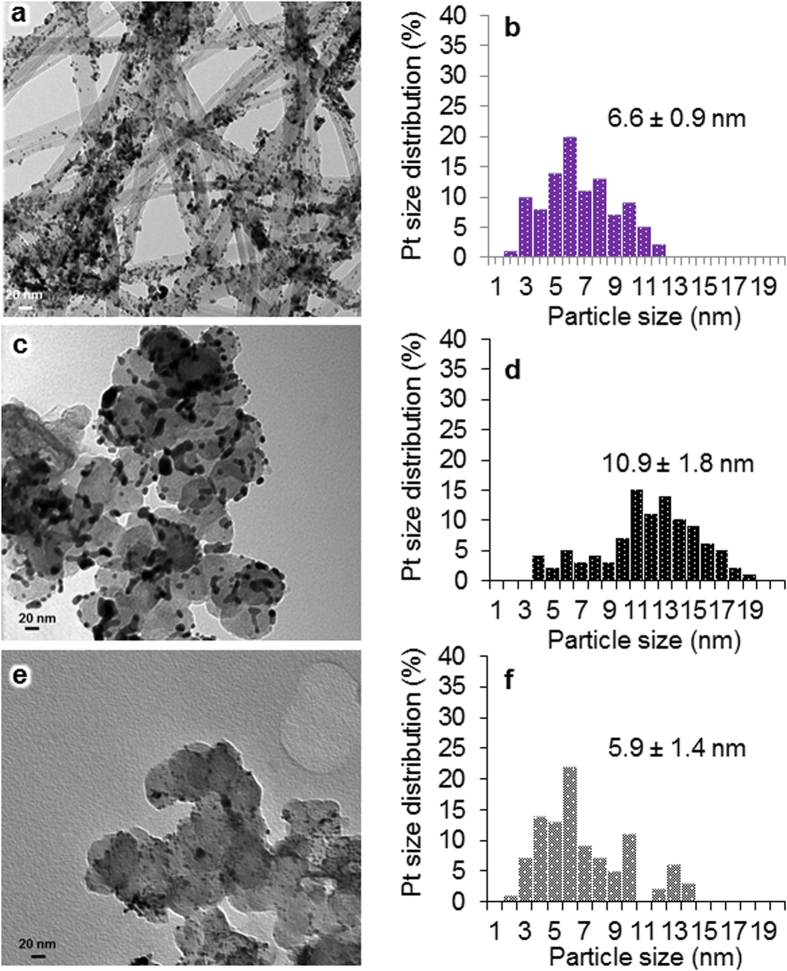
TEM images and Pt-size distribution after durability tests. TEM images and Pt-size distribution of MWNT/PyPBI/Pt/Nafion electrocatalyst (**a**,**b**, respectively), CB/PyPBI/Pt/Nafion electrocatalyst (**c**,**d**, respectively), and CB/Pt/Nafion-treated electrocatalyst (**e**,**f**, respectively), in which the samples were collected from the cathode side after the durability test.

**Figure 8:**
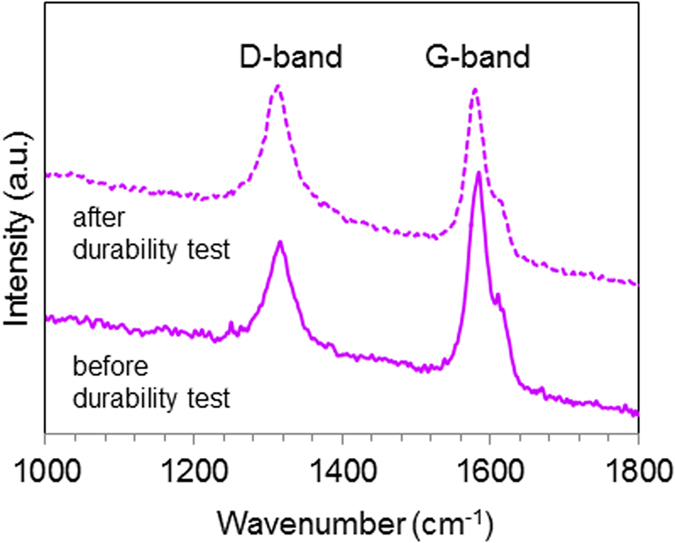
Raman spectra. Raman spectra of the MWNT/PyPBI/Pt/Nafion before (solid purple) and after (dashed purple) durability testing.

The Nafion ionomer was stably coated as the outer layer of the MWNT/PyPBI/Pt and CB/PyPBI/Pt by the aid of PyPBI through the acid-base reaction to form a double-coating layer as indicated by the XPS analysis (see Fig. 2). Although the two composites possess similar double-coating structure, the EIS analysis of the MWNT/PyPBI/Pt/Nafion-based MEA materials showed a much lower total ohmic resistance probably due to the fibrous network structure of the conductive MWNTs. Accordingly, a higher FC performance was observed for the MWNT/PyPBI/Pt/Nafion-based MEA (see Fig. 4b). Most importantly, the MEA fabricated using the MWNT/PyPBI/Pt/Nafion electrocatalyst showed a remarkably high durability with only a 5% loss of the initial FC potential at 200 mA/cm^2^ after 500,000 potential cycles, which showed a much higher stability compared to commercial CB/Pt-based MEA that exhibited a 50% potential loss at 200 mAcm^–2^ after only 10,000 potential cycles (Fig. 7). We propose three possible reasons that enabled such a remarkable durability for the MWNT/PyPBI/Pt/Nafion-based MEA; namely, (i) the MWNTs have a highly crystalline graphitic structure compared to CB as we observed in the Raman data before and after the durability test (see Fig. 8), (ii) PyPBI that wrapped MWNTs functioned to prevent the loss of the Pt-NPs through the interaction between the PyPBI and Pt-NPs during the durability test as indicated in Fig. 7, and (iii) formed stable double-polymer-coating structure served to remain a high proton conductivity for long, leading to a high performance and durability.

In conclusion, the present study provides a promising material design for the next generation PEMFC since such a high durability is strongly demanded from industry. We believe that the replacement of the conventional CB-based FC catalyst that is currently used in homes and automobiles with the presented catalyst would be an advantageous step since the total performance of the present catalyst surpasses that of the conventional ones.

## Methods

### Materials

*N,N*-dimethylacetamide, ethylene glycol (EG), hydrogen hexachloroplatinate hexahydrate (H_2_PtCl_6_·6H_2_O), 2-propanol, methanol, sulfuric acid and nitric acid were purchased from Wako Pure Chemical, Ltd., and used as received. Nafion solution (5 wt%) and Nafion® 117 membrane were purchased from Sigma-Aldrich. Multi-walled carbon nanotubes (MWNTs; ca. 20-nm diameter) were kindly supplied from the Nikkiso Co. Carbon black (Vulcan XC-72R) was purchased from Cabot Chemical Co., Ltd. Poly[2,2’-(2,6-pyridine)-5,5′-bibenzimidazole] (PyPBI) (Fig. 1a) was synthesized according to a previously reported method^40^. The MWNT/PyPBI, CB/PyPBI, CB/PyPBI/Pt and MWNT/PyPBI/Pt composites were prepared according to our previous reports^12^,^14^.

### Synthesis of MWNT/PyPBI/Pt/Nafion and CB/PyPBI/Pt/Nafion composites

Ten mg of the MWNT/PyPBI/Pt composite was added to an aqueous solution of 2-propanol (80 vol%) (20 mL), which was sonicated using a bath-type sonicator (BRANSON 5510) for 5 min to disperse the composite material, to which 66 μL of a Nafion solution (5 wt%) that was diluted in a 2-propanol solution (5 mL) was added. The resulting mixture was stirred for 1 h at room temperature, followed by filtration and rinsing using 2-propanol/methanol to remove any excess physically-attached Nafion. Finally, the obtained composite (MWNT/PyPBI/Pt/Nafion) was vacuum dried. By the same process, the CB/PyPBI/Pt/Nafion composite was prepared by replacing the MWNTs with CB.

### Synthesis of CB/Pt (Nafion-treated)

The CB/Pt composite material (10 mg) was ultrasonically dispersed in an aqueous 2-propanol solution (20 mL) containing 66 μL of a Nafion solution (5 wt%). The resulting mixture was stirred for 1 h at room temperature followed by filtration and rinsing with a 2-propanol/methanol solvent. Finally, the obtained composite was vacuum dried.

### Materials characterization

IR spectra were recorded using a Perkin-Elmer Spectrum-65 FT-IR spectrometer in the wavenumber range of 4000–700 cm^−1^ by accumulating 4 scans at a 4 cm^−1^ resolution. The XPS spectra were measured using an AXIS-ULTRADLD (Shimadzu, KRATOS) instrument. The Raman spectroscopy measurement was carried out using a Raman RXN Systems (Kaiser, excitation, 785 nm) at room temperature. The TGA measurements were obtained by a TGA-50 (Shimadzu) at the heating rate of 10 °C/min under 20 mL/min flowing air. The TEM micrographs were obtained using an electron microscope (JEM-2010) at the acceleration voltage of 120 kV. A copper grid with a carbon support (Okenshoji) was used for the TEM observations. SEM micrographs were obtained using SU8000 (Hitachi High-tech). Electrochemical Impedance Spectroscopy (EIS) measurements were carried out in the frequency range of 100 kHz ~0.1 Hz using a Solartron 1287/1260 potentiostat/frequency response analyzer equipped with Zplot software (Zview, Scribner Associate, Inc.). The Nyquist spectra were used for the analysis. The obtained data were fitted using the software.

### Electrochemical measurements

The electrochemical measurements were performed using a rotating ring disk electrode (RRDE-3, Bioanalytical Systems, Inc.) with a conventional three-electrode configuration at room temperature. A glassy carbon electrode (GC) with the geometric surface area of 0.282 cm^2^ was used as the working electrode. A Pt wire and Ag/AgCl were used as the counter and reference electrodes, respectively. The Ag/AgCl reference electrode was calibrated against the reference hydrogen electrode (RHE) potential in 0.1 M HClO_4_. All working potentials are indicated versus the RHE. The potential of the sample electrode was controlled by a potentiostat (Model DY2323, ALS). The catalyst solution of the prepared composites was prepared as follows: The composite material (1.0 mg) was ultrasonically dispersed in an aqueous EG solution (2.0 mL) to provide a homogeneous suspension. A portion of the composite suspension was then cast on a GC electrode to produce a load of 14 μg_Pt_/cm^2^. Finally, the cast GC electrode was air-dried. For the commercial CB/Pt electrocatalyst, the characterization process did not show any Nafion content in the completed composites. Thus, the same amount of the Nafion ionomer found in the CB/PyPBI/Pt/Nafion-treatment was added to the GC-based electrode films to obtain comparable results. The CVs were carried out at a scan rate of 50 mV/sec in a nitrogen-saturated 0.1 M HClO_4_ solution to determine the ECSAs of the electrocatalysts.

### MEA fabrication and fuel cell performance measurements

The electrocatalyst composite material was ultrasonically dispersed in an aqueous EG solution, then deposited on a gas diffusion layer (GDL) (SIGRACET gas diffusion media GDL 25 BC, SGL Carbon Group) by vacuum filtration. The GDL was used as a filter to obtain a gas diffusion electrode (GDE)^14^. As already described, the characterization did not show any Nafion content in the commercial CB electrocatalyst. Thus, the same amount of the Nafion ionomer found in the CB/PyPBI/Pt/Nafion composite was cast on the GDE. The Nafion membrane was then sandwiched between two GDEs (1 cm^2^) of each electrocatalyst to fabricate the two different MEAs (Supplementary Fig. 1). The performance of the assembled MEAs was measured at 70 °C using a computer-controlled fuel cell test system (model 890e, Scribner Associate, Inc.). The polarization curves were recorded at atmospheric pressure in hydrogen (100 mL/min flow rate) and air (200 mL/min flow rate) as the anode and cathode gases, respectively, under a 50% relative humidity (RH).

### Durability test

The assembled MEA was subject to an accelerating durability test based on the protocol provided by the Fuel Cell Commercialization of Japan (FCCJ)^41^. Typically, the potential sweeps were cycled between 1.0 and 1.5 V at 70 °C under a 50% humidified condition. The scanning rate was 0.5 V/s. H_2_ and N_2_ were fed to the anode and cathode, respectively. The I-V curves were recorded every 1,000 cycles after switching the cathode gas from N_2_ to air (Supplementary Fig. 2). The potential was plotted as a function of the cycle number at 200 mA/cm^2^ (this current density is often used for stationary PEMFCs^37^).

## Additional Information

**How to cite this article**: Berber, M. R. *et al.* A highly durable fuel cell electrocatalyst based on double-polymer-coated carbon nanotubes. *Sci. Rep.* **5**, 16711; doi: 10.1038/srep16711 (2015).

## Supplementary Material

Supplementary Information

## Figures and Tables

**Table 1:** Data analysis of the measured impedance spectra.

	CNT- basedMEA	CB-basedMEA
R_Ω_ (mOhm cm^2^)	201	285
R_ct_ (mOhm cm^2^)	32	287
R_g_ (mOhm cm^2^)	532	689
Total R_ct_ and R_g_(mOhm cm^2^)	564	976
