# Supplementary material for: A highly durable fuel cell electrocatalyst based on double-polymer-coated carbon nanotubes
Source: Sci Rep. 2015 Nov 23;5:16711. doi: 10.1038/srep16711 (PMC4655398; doi:10.1038/srep16711)
Supplement: Supplementary Information [file srep16711-s1.docx]

**Supplementary Information**

A highly durable fuel cell electrocatalyst based on double-polymer-coated carbon nanotubes

Mohamed R. Berber^1,2^, Inas H. Hafez^1,3^ Tsuyohiko Fujigaya^1,4^ and Naotoshi Nakashima*^1,4,5^

^1^International Institute for Carbon Neutral Energy Research (WPI-I2CNER), Kyushu University, 744 Motooka, Nishi-ku, Fukuoka 819-0395 (Japan)

^2^Department of Chemistry, Faculty of Science, Tanta University, Tanta 31527 (Egypt)

^3^Department of Natural resources and Agricultural Engineering, Faculty of Agriculture, Damanhour University, Damanhour 22516 (Egypt)

^4^Department of Applied Chemistry, Graduate School of Engineering, Kyushu University, 744 Motooka, Nishi-ku, Fukuoka 819-0395 (Japan)

^5^JST-CREST, 5 Sanbancho, Chiyoda-ku, Tokyo, 102-0075 (Japan)

*e-mail: nakashima-tcm@mail.cstm.kyushu-u.ac.jp

**a**

**b**

**Supplementary Figure 1a,b∣Schematic illustrations.** Schematic illustration for the preparation of double polymer coating of MWNT/PyPBI/Pt/Nafion **(a)**, and CB/PyPBI/Pt/Nafion **(b).**


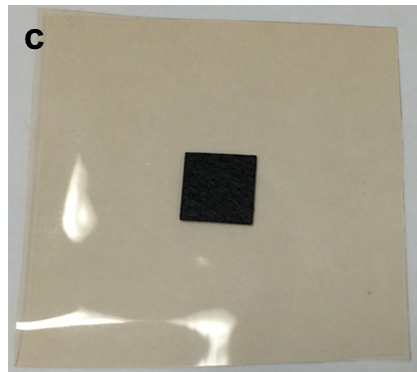


**Supplementary Figure 1c∣Membrane electrode assembly.** Photo image illustrates the dimensions of the MEA used in the study.


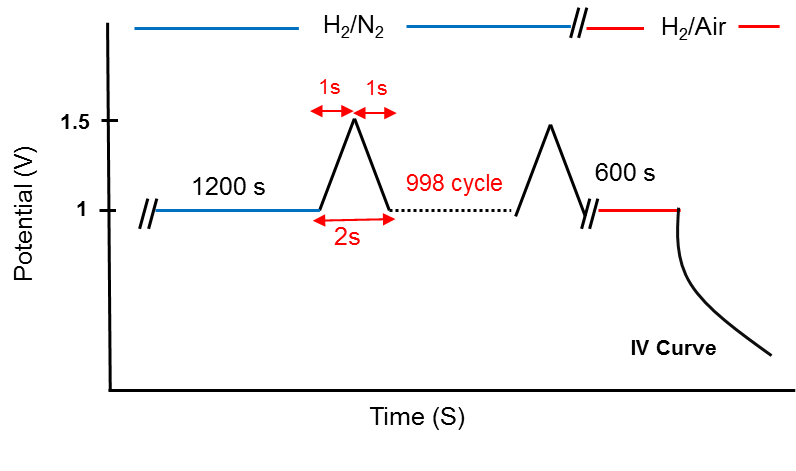


**Supplementary Figure 2∣Durability protocol.** A schematic illustration of the potential protocol used in the durability test.


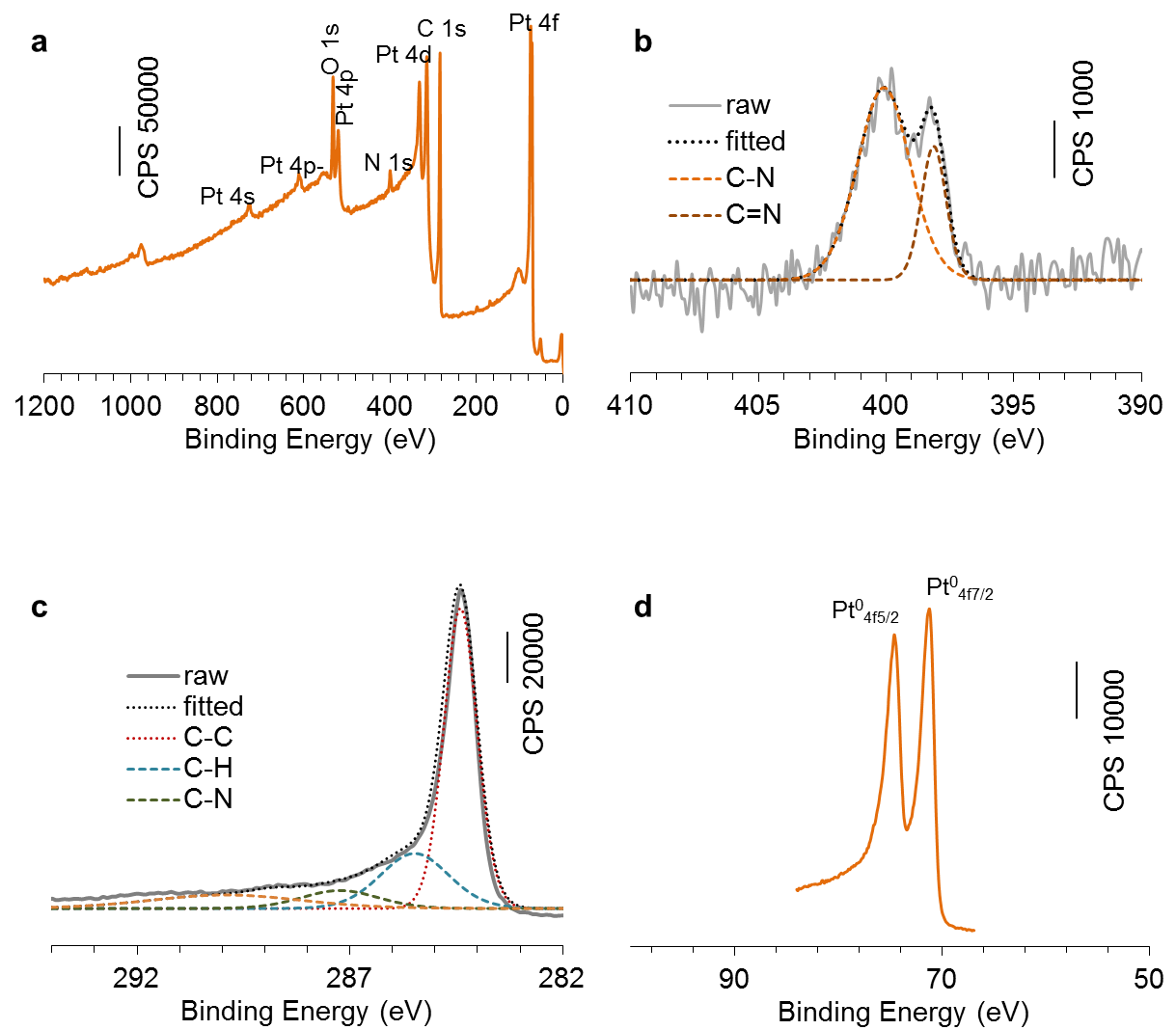


**Supplementary Figure 3∣XPS analysis. (a)** XPS survey spectrum of the MWNT/PyPBI/Pt. **(b)** XPS core spectrum of the N_1s_ and its fitted components. **(c)** XPS of the C_1s_ and its fitted components. **(d)** XPS of the Pt_4f_.

*Detailed explanation of* ***Supplementary Figure 3:***  Supplementary Figure 3a shows a survey scan of the MWNT/PyPBI/Pt composite. The N_1s_ core (Fig. S3b) shows a doublet peak at 398.2 and 400.2 eV, corresponding to the double- and single-bonded nitrogen on the PyPBI,[^1^](#_ENREF_1) indicating the wrapping of the MWNTs by the PyPBI. The N/C ratio determined from the XPS was 5/95, indicating the presence of a thin layer of the PyPBI around the MWNTs. The C_1s_ core-level spectrum (Supplementary Figure 3c) shows four major sources of carbon peaks at 284.5 eV, 285.6 eV, 287.5 eV and 291.2 eV which are attributed to C-C, C-H, C-N of PBI and the π-π bonding of MWNTs. The Pt_4f_ doublet peaks at 71.1 and 74.4 eV (Supplementary Figure 3d) are attributed to Pt^0^_4f7/2_ and Pt^0^_4f5/2_, respectively, [^2^](#_ENREF_2) indicating the successful reduction process of Pt metal onto the surface of the MWNT/PyPBI composite. Similar results were observed in the case of CB/PyPBI/Pt composite (see Supplementary Figure 4).


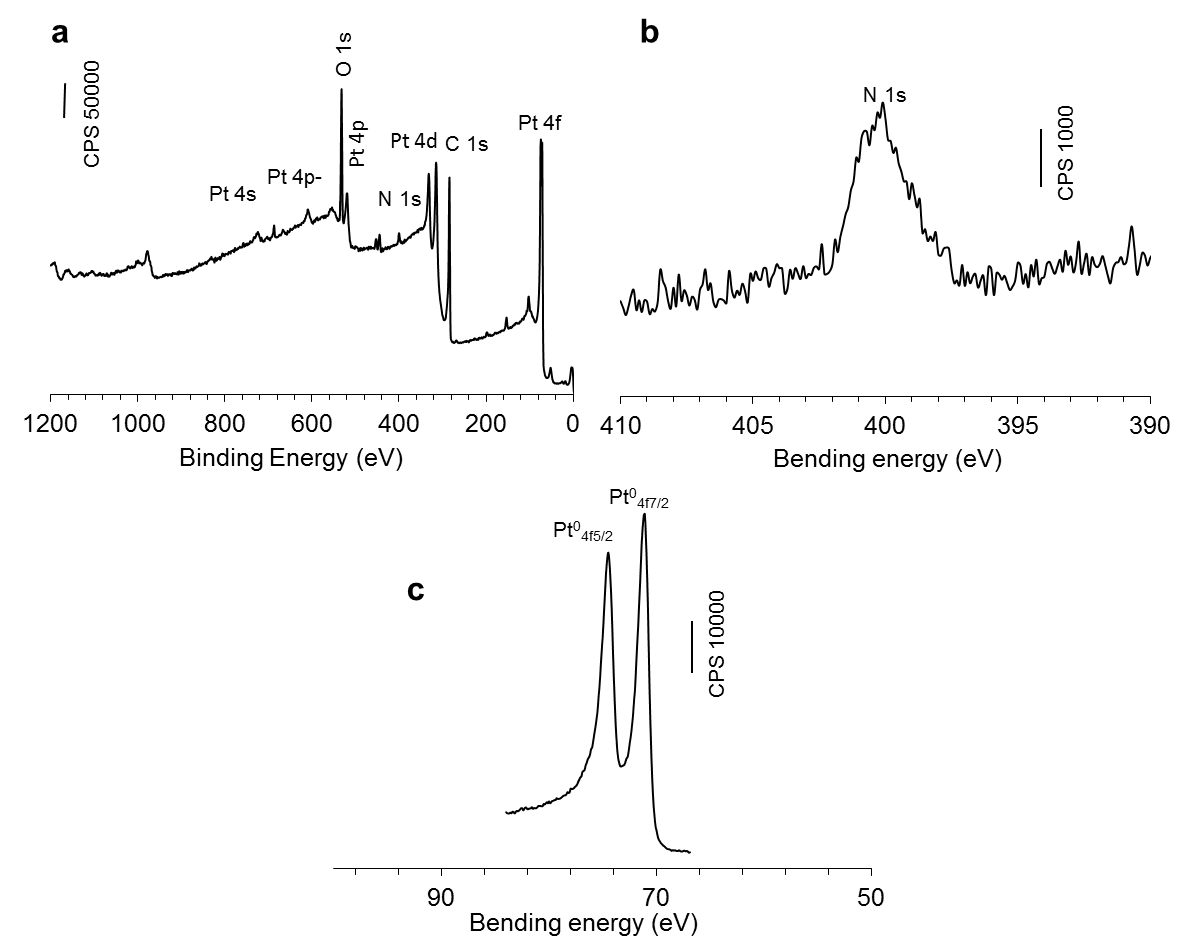


**Supplementary Figure 4∣XPS analysis.** XPS survey spectrum of the CB/PyPBI/Pt composite **(a)**. XPS core spectrum of: N_1s_ **(b)**, and Pt_4f_ **(c)**.


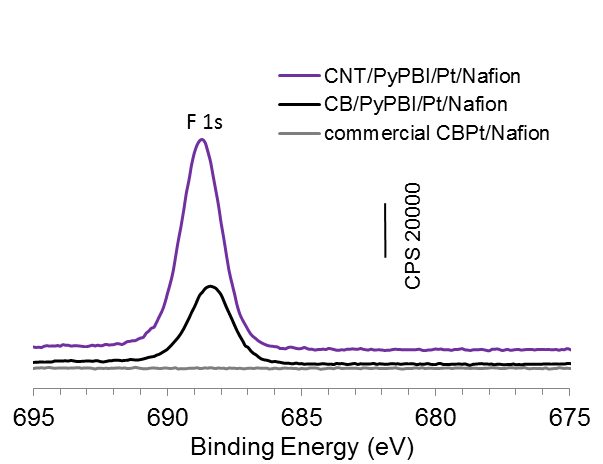


**Supplementary Figure 5∣XPS analysis.** XPS core spectrum of F_1s_ after Nafion treatment of: MWNT/PyPBI/Pt (purple spectrum), CB/PyPBI/Pt (black spectrum) and CB/Pt (gray spectrum).


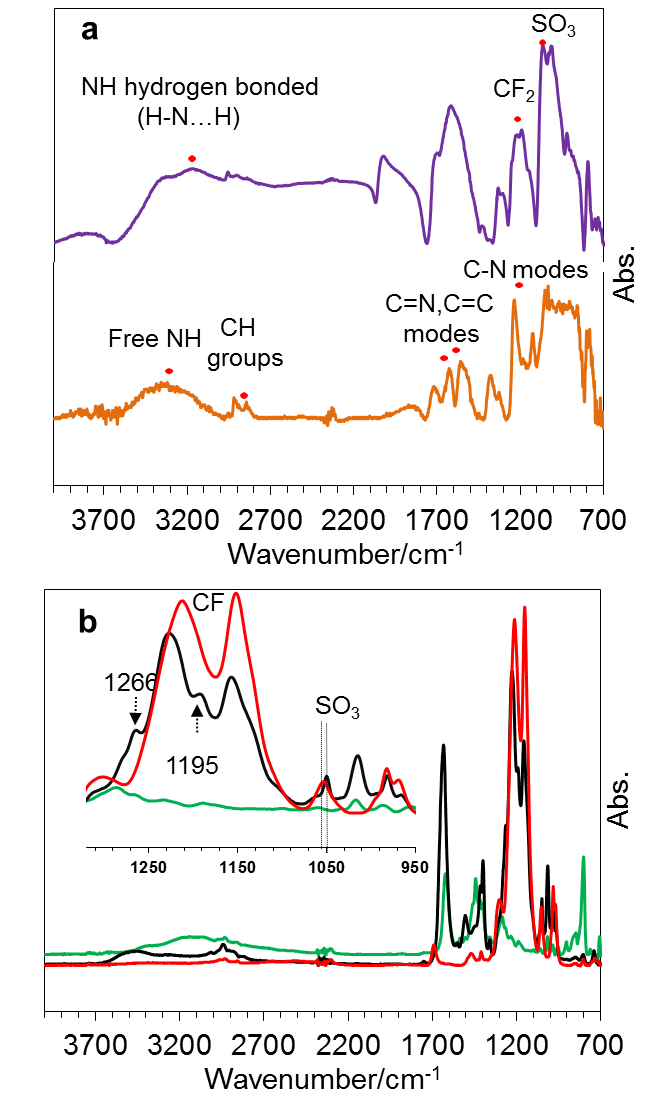


**Supplementary Figure 6∣** **IR measurement.** **(a)** IR spectra of MWNT/PyPBI/Pt before (orange) and after (purple) Nafion coating. **(b)** IR spectra of Nafion polymer (red), PyPBI polymer (green) and Nafion-PyPBI composite (black). The inset of Supplementary Figure 6b shows the magnification of the low wave number region.

*Detailed explanation of* ***Supplementary Figure 6 and References:***

Supplementary Figure 6a shows the IR spectra of the MWNT/PyPBI/Pt before (orange) and after (purple) the Nafion-coating. As can be seen in the spectra, both the characteristic bands of PyPBI[^3^](#_ENREF_3)^,^ [^4^](#_ENREF_4) and Nafion[^5^](#_ENREF_5)^,^ [^6^](#_ENREF_6) are present. Unfortunately, due to the strong background of the MWNTs, detailed information about the interaction between the Nafion and PyPBI was not obtained. Thus, a composite of only PyPBI and Nafion was separately prepared using a similar method for the preparation of the MWNT/PyPBI/Pt/Nafion. As displayed in Fig. S6b, the Nafion spectrum (blue) showed the antisymmetric vibrations of the CF bonds at 1150 and 1209 cm^-1^. The doublet band at 971 and 983 cm^-1^, and the band at 1057 cm^-1^, which are the characteristic bands of the side chain of Nafion, are assigned to the symmetric vibrations of the C-O-C bonds and SO stretching vibration of the sulphonic groups, respectively.[^5^](#_ENREF_5)^,^ [^6^](#_ENREF_6) The PyPBI spectrum (orange) also showed the characteristic bands of polybenzimidazole as previously reported.[^3^](#_ENREF_3)^,^ [^4^](#_ENREF_4)^,^ [^7^](#_ENREF_7) The stretching vibration bands of the C=C aromatic, CN, CH and NH groups were detected at 1443 cm^-1^, 1620 cm^-1^, 2880-2990 cm^-1^ and 3000-3450 cm^-1^, respectively.

The IR spectrum of the Nafion-PyPBI composite (purple) showed similar bands to those derived from both Nafion and PyPBI; however, in the composite state, we observed a shift in the sulphonic band of Nafion towards a lower wavenumber (see the inset of Supplementary Figure 6b). This shift reflects a distortion in the symmetric structure of the SO_3_^-^ group, which should be directly related to the change in the SO_3_^-^ surface charge that results from a chemical bonding. This shift was consistent with the band displacement reported by Tannenbaum et al. [^8^](#_ENREF_8) for the interaction between the Nafion-SO_3_^-^ and poly(ethylacrylate-*co*-4-vinyl pyridine). They explained this shift by a lower polarization of the S-O dipole due to an increased separation between the SO_3_^-^ and H^+^ following the proton transfer between the sulfonate groups and positively charged pyridine units.

The CF stretching vibration bands at 1150 and 1209 cm^-1^ were also affected by this distortion and became more broadened.[^9^](#_ENREF_9) Also, of importance is the NH stretching band in the region of 3000-3450 cm^-1^. Namely, the NH band showed a broadening after the Nafion-PyPBI coating, which is usually the result of a hydrogen bonding interaction (protonation of free NH groups).[^7^](#_ENREF_7) Additionally, it is possible that the bands that appeared at 1195 and 1266 cm^-1^ are the result of a bonding interaction between the SO_3_^-^ group of Nafion and the protonated NH group of PyPBI.[^9^](#_ENREF_9) Based on the results of XPS and IR, it is therefore reasonable to conclude that Nafion interacts ionically with PyPBI, forming doubly-polymer-coated MWNTs

**References**

1. Hamilton, L.E., Sherwood PMA, Reagan BM. X-Ray Photoelectron-Spectroscopy Studies of Photochemical Changes in High-Performance Fibers. *Appl. Spectrosc.* **47**, 139-149 (1993).

2. Tian ZQ, Jiang SP, Liang YM, Shen PK. Synthesis and Characterization of Platinum Catalysts on Multiwalled Carbon Nanotubes by Intermittent Microwave Irradiation for Fuel Cell Applications. *J. Phys. Chem. B* **110**, 5343-5350 (2006).

3. Suryani, Chang C-M, Liu Y-L, Lee YM. Polybenzimidazole membranes modified with polyelectrolyte-functionalized multiwalled carbon nanotubes for proton exchange membrane fuel cells. *J. Mater. Chem.* **21**, 7480-7486 (2011).

4. Maity S, Jana T. Soluble Polybenzimidazoles for PEM: Synthesized from Efficient, Inexpensive, Readily Accessible Alternative Tetraamine Monomer. *Macromolecules* **46**, 6814-6823 (2013).

5. Hobson LJ, Ozu H, Yamaguchi M, Hayase S. Modified Nafion 117 as an Improved Polymer Electrolyte Membrane for Direct Methanol Fuel Cells. *J. Electrochem. Soc.* **148**, A1185-A1190 (2001).

6. Luo Q, Zhang H, Chen J, You D, Sun C, Zhang Y. Preparation and characterization of Nafion/SPEEK layered composite membrane and its application in vanadium redox flow battery. *J. Membrane Sci.* **325**, 553-558 (2008).

7. Musto P, Karasz FE, MacKnight WJ. Fourier transform infra-red spectroscopy on the thermo-oxidative degradation of polybenzimidazole and of a polybenzimidazole/polyetherimide blend. *Polymer* **34**, 2934-2945 (1993).

8. Tannenbaum R, Rajagopalan M, Eisenberg A. Fourier transform infrared studies of ionic interactions in perfluorinated acid copolymer blends. *J. Polym. Sci., Part B: Polym. Phys.* **41**, 1814-1823 (2003).

9. Peak D, Ford RG, Sparks DL. An in Situ ATR-FTIR Investigation of Sulfate Bonding Mechanisms on Goethite. *J. Colloid Interface Sci.* **218**, 289-299 (1999).


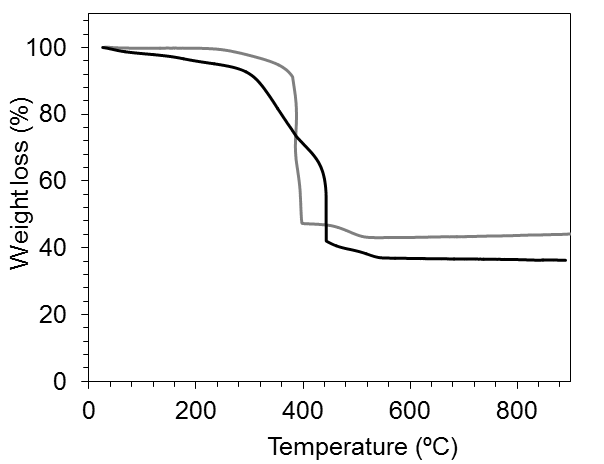


**Supplementary Figure 7∣** **TGA analysis.** TGA curves of CB/PyPBI/Pt: before (gray) and after (black) Nafion coating.

Calculation of the real Nafion content in the prepared composites

The amount of the Nafion in the composite was calculated by considering that the amount of Pt in the composite did not change after the addition of Nafion ionomer. Thus, for each 1 mg of the composite, X mg of Nafion was found.

Hence,

*For MWNT/PyPBI/Pt/Nafion composite*

[Pt ratio before Nafion coating = Pt ratio after Nafion coating; which means 44.1 wt% = (1mg+X) x 38 wt%]. Therefore, X (real Nafion content in the composite) equals 0.16 mg for each 1mg of composite before Nafion addition.

By the same process the real amount of Nafion onto *CB/PyPBI/Pt composite* was calculated to be 0.146 mg for each 1mg of composite before Nafion addition.


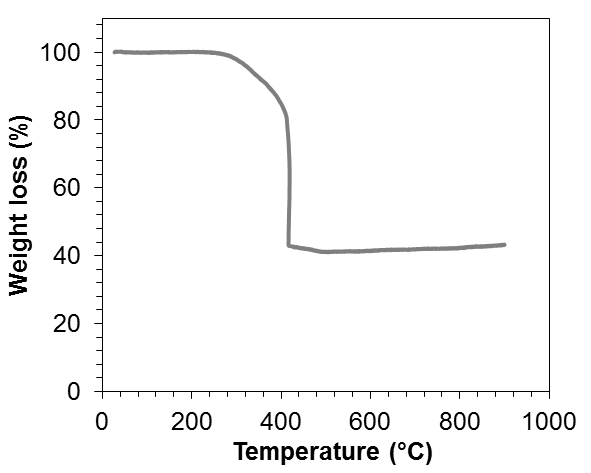


**Supplementary Figure 8∣** **TGA analysis.** TGA curve of CB/Pt composite.


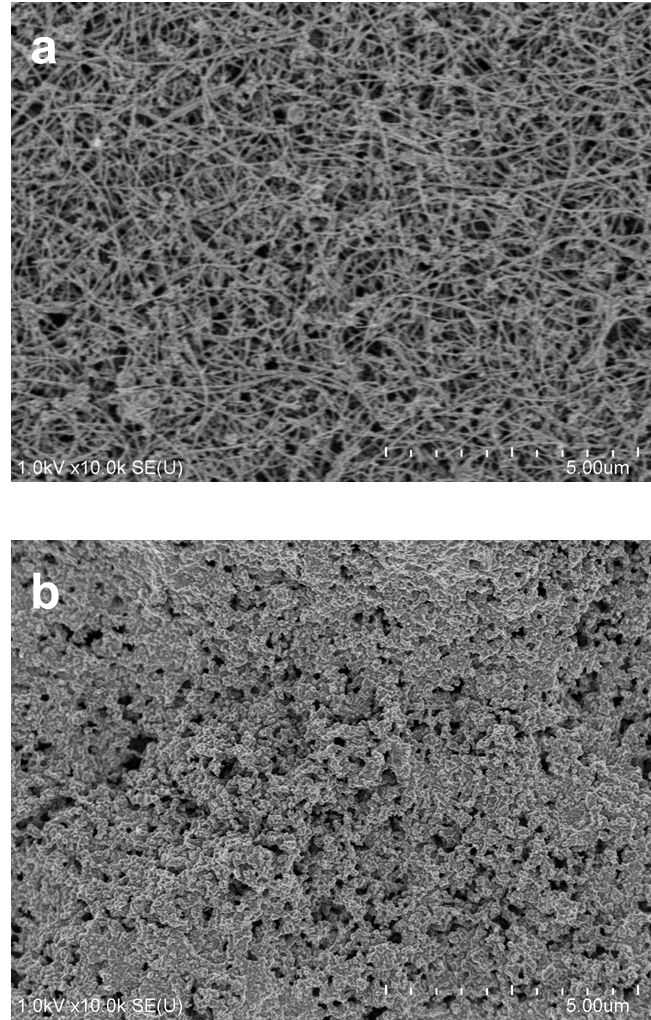


**Supplementary Figure 9∣** **SEM analysis.** SEM image of gas diffusion electrode of: (**a**) polymer-wrapped MWNTs, and (**b**) polymer-wrapped CB.


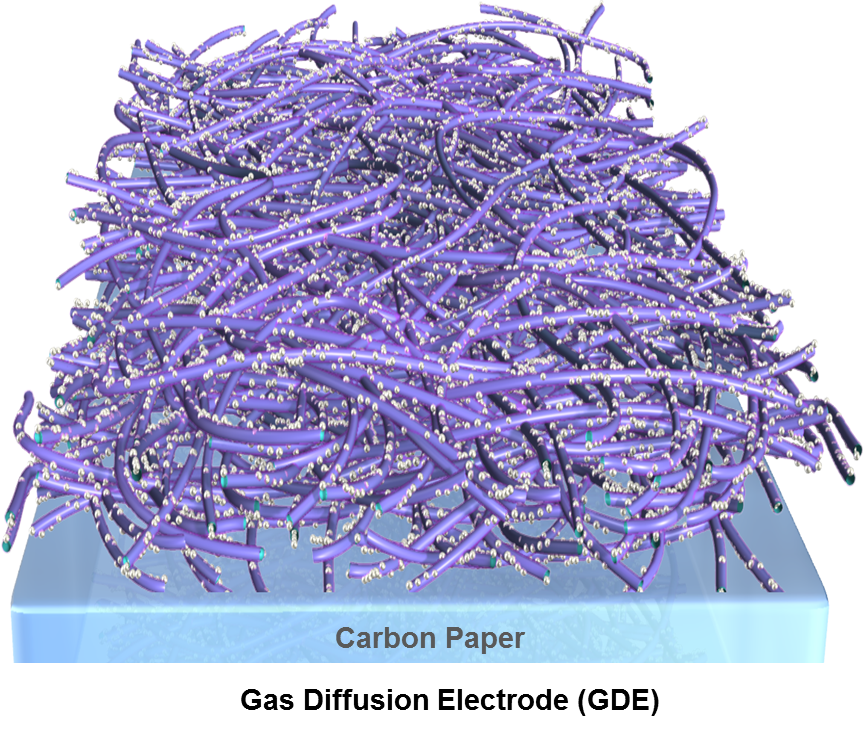


**Supplementary Figure 10∣** **Schematic illustration.** The morphology of the gas diffusion electrode of the MWNT/PyPBI/Pt/Nafion catalyst.


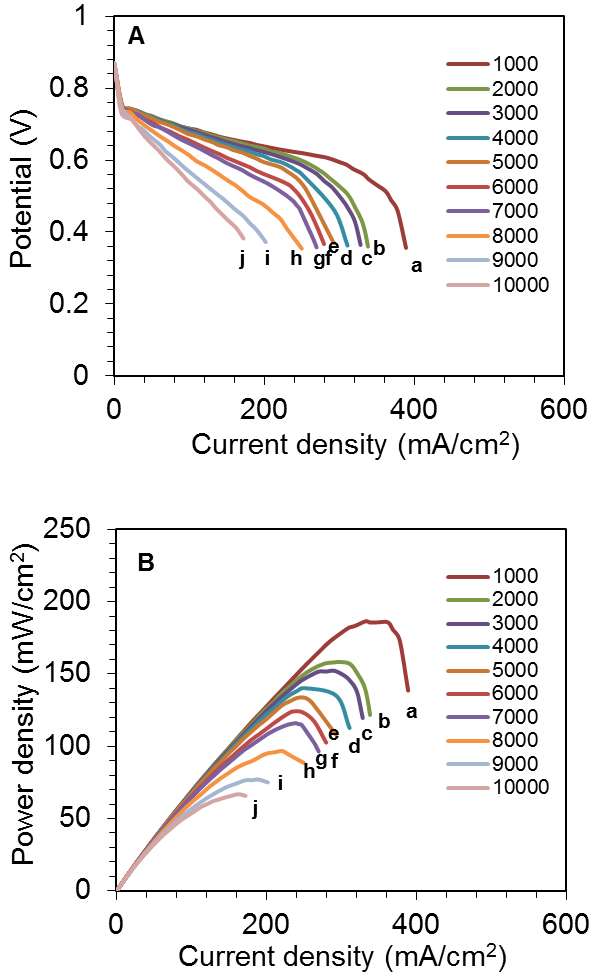


**Supplementary Figure 11∣Durability results.** Polarization curves **A**, and power density curves **B**, during durability testing of commercial CB/Pt-based MEA. The Arabic letters a, b, c, d, e, f, g, h, I, and j represent the performance after 1000, 2000, 3000, 4000, 5000, 6000, 7000, 8000, 9000, and10,000 potential cycles.


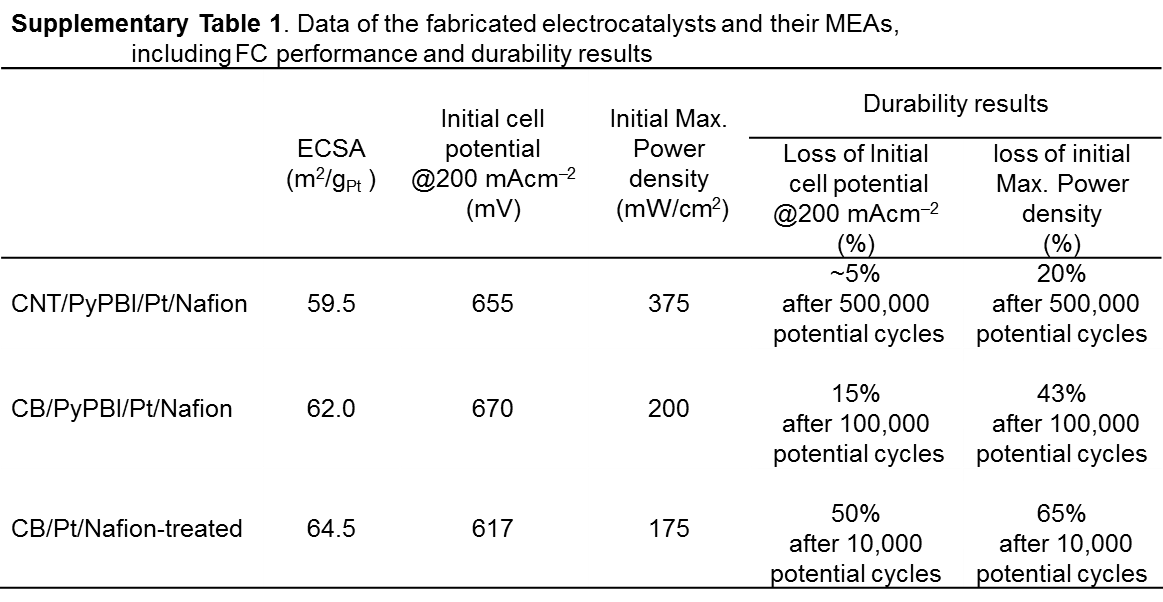


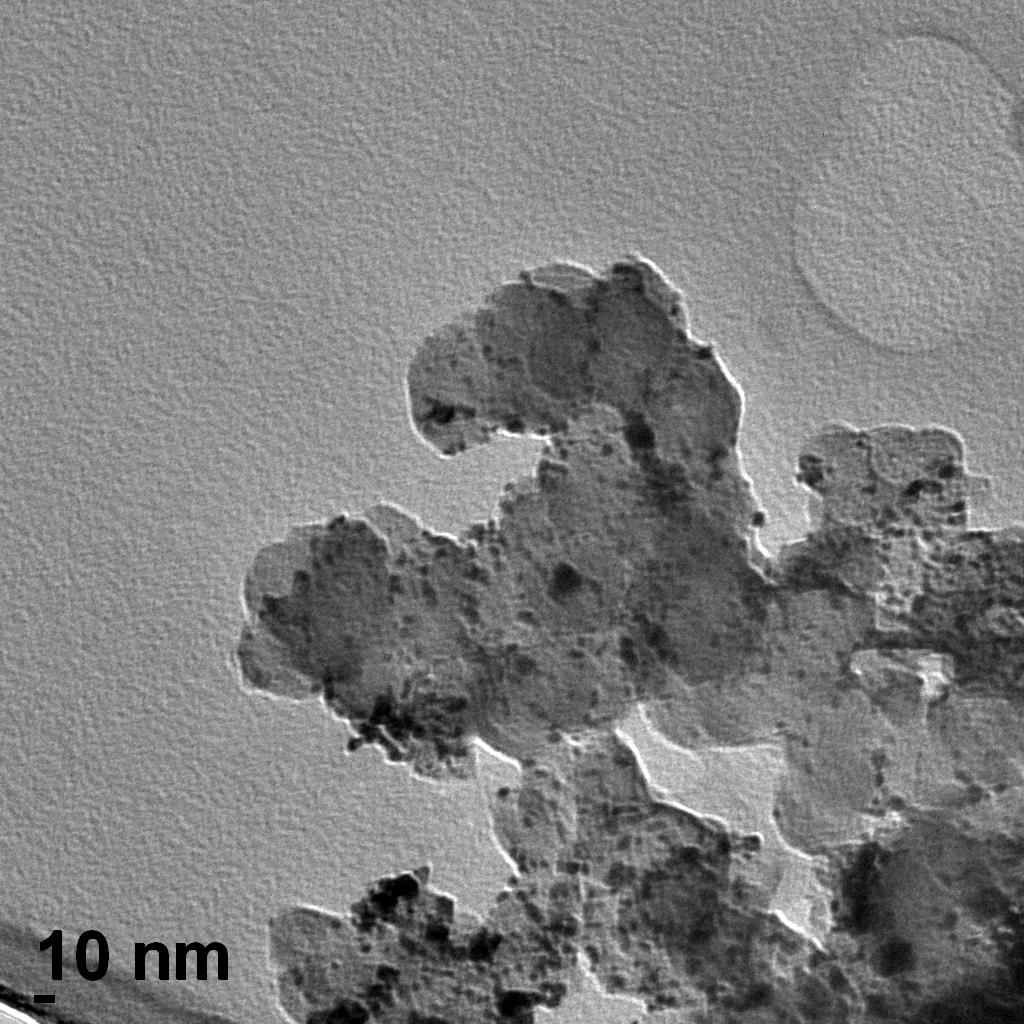


**Supplementary Figure 12∣** **TEM analysis.** TEM image of the commercial CB/Pt electrocatalyst after the durability test.
